# The protective effect of COVID-19 vaccines on developing multisystem inflammatory syndrome in children (MIS-C): a systematic literature review and meta-analysis

**DOI:** 10.1186/s12969-023-00848-1

**Published:** 2023-08-07

**Authors:** Mohamad Hamad Saied, Laura van der Griend, Joeri W van Straalen, Nico M. Wulffraat, Sebastiaan Vastert, Marc H A Jansen

**Affiliations:** 1 Department of Paediatric Immunology and Rheumatology, Wilhelmina Children’s Hospital, University Medical Center Utrecht, P.O. box 85090, Utrecht, 3508 AB The Netherlands; 2 Department of Pediatrics, Carmel Medical Center, Technion Faculty of Medicine, Haifa, Israel; 3 University Medical Center Utrecht, Utrecht, The Netherlands; 4 Center for Translational Immunology, University Medical Center Utrecht, Utrecht, The Netherlands

**Keywords:** MIS-C, PIMS-TS, COVID-19, mRNA vaccine

## Abstract

**Objective:**

To review whether the current COVID-19 vaccines can prevent the occurrence of multisystem inflammatory syndrome in children (MIS-C) and adolescents.

**Methods:**

A systematic literature review and meta-analysis were performed. The data were abstracted following the Preferred Reporting Items for Systematic Reviews and Meta-Analyses (PRISMA) guidelines. Primary outcome was the efficacy of COVID-19 vaccination in preventing MIS-C development. The search was performed in PubMed and Embase.

**Results:**

The review yielded 13 studies, which were included for critical appraisal and data extraction. The available studies showed a reduced incidence of MIS-C after mRNA COVID-19 vaccination in children aged 12–18 years. Four studies were eligible for meta-analysis and the pooled odds ratio for MIS-C in vaccinated children compared to unvaccinated children was 0.04 (95% confidence interval: 0.03–0.06). Additionally, the risk of MIS-C as an adverse effect of vaccination was much lower compared to the risk of MIS-C post-infection.

**Conclusions:**

Our systematic review highlights the current available evidence on the efficacy of COVID-19 vaccination in preventing MIS-C. The published studies so far – mainly conducted during the Delta wave – indicate that (original strain) COVID-19 mRNA vaccines in children are safe and associated with significantly less development of MIS-C. These findings further reinforce the recommendation for COVID-19 vaccination in children, which should be promoted and largely supported.

**Supplementary Information:**

The online version contains supplementary material available at 10.1186/s12969-023-00848-1.

## Background

Coronavirus disease 2019 (COVID-19) mRNA vaccines demonstrated safety, immunogenicity, and efficacy against severe acute respiratory syndrome coronavirus 2 (SARS-CoV-2) infection in adults, adolescents (12–18 years) and children (5–11 years), and were therefore authorized by the Food and Drug Administration (FDA) and at the end of 2021 by the European Medicine Agency (EMA) for use in children aged 5–11 years [1–3]. There has been debate on whether children should be vaccinated, as the pediatric population generally shows a milder and non-fatal course of COVID-19 compared to adults [4]. However, in some children, COVID-19 can have severe consequences. There has been an increasing number of reported cases of pediatric inflammatory multisystem syndrome temporally associated with SARS-CoV-2 (PIMS-TS), also called multisystem inflammatory syndrome in children (MIS-C). This severe hyperinflammatory syndrome, which shares some similarities with Kawasaki disease and some unique clinical features with serious complications, occurs approximately three weeks (range 2–6 weeks) after SARS-CoV-2 infection [5]. It is defined by the Centers for Disease Control and Prevention (CDC) as a severe illness requiring hospitalization in individuals aged < 21 years, with fever for ≥ 24 h, elevated inflammatory markers with multisystem (≥ 2) organ involvement and a suspected or confirmed SARS-CoV-2 infection within the last four weeks [6]. Even though MIS-C and Kawasaki disease show phenotypic similarities, there are important differences regarding morbidity and mortality. MIS-C is more associated with gastrointestinal symptoms and cardiovascular system involvement [7]. Cardiac involvement, including diminished ventricular function, coronary artery dilatation and diffuse myocardial oedema, are more prevalent and severe in MIS-C than in Kawasaki disease and cardiogenic shock is observed in MIS-C in 40–80% of the patients compared to 2–7% in Kawasaki [7, 8]. Additionally, more than 60% of the MIS-C patients are admitted to the pediatric intensive care unit (PICU) [7–9]. Mortality rates for MIS-C are estimated to be around 1% in high-income countries, but significantly higher in developing countries (reported up to 9%)[10] and overall higher thanin Kawasaki disease, which has a mortality rate of 0.1–0.3% in patients treated with intravenous immunoglobulin (IVIG) [11, 12]. MIS-C patients are currently treated with IVIG, glucocorticosteroids, aspirin, supportive treatments, and refractory cases with additional biological DMARDs [13].

As of May 2022, a total of 8,525 MIS-C patients have been reported in the United States, of whom 69 patients have died [14]. Available estimates of cases worldwide are lacking. Most MIS-C patients were healthy children, not known to have an underlying medical disorder at time of infection and MIS-C. Until now, the pathophysiology of MIS-C remains largely unknown [15], but it has been hypothesized that MIS-C is a dysregulated immune response characterized by a cytokine storm associated with a superantigen-like activation of T-cells [16–19].

Currently, it is not clear if available COVID-19 vaccines are able to prevent these serious complications of SARS-CoV-2 infection in children. The purpose of this paper is therefore to perform a systematic literature review and meta-analysis on whether current COVID-19 vaccines have a protective effect on the development of MIS-C in children and adolescents aged 5–18 years.

## Methods

### Literature search and data extraction

We conducted a systematic review of the literature on the protective effect of the COVID-19 vaccine on developing multisystem inflammatory syndrome in children (MIS-C). The systematic review protocol was designed in accordance with the Preferred Reporting Items for Systematic reviews and Meta-Analysis (PRISMA) [20]. The research question was: “does the COVID-19 vaccine have a protective effect on developing multisystem inflammatory syndrome in children (MIS-C)?”. The primary endpoint was efficacy and the secondary endpoint was safety. Efficacy was defined as the capacity of COVID-19 vaccines to prevent the development of MIS-C and safety by evaluating the data on COVID-19 vaccination as a trigger for MIS-C development.

The research question was adapted into search terms according to the PICO method (patient-intervention-comparison-outcome). Participants: children and adolescents 5–18 years old; intervention: COVID-19 vaccination; comparison: children who did not receive a COVID-19 vaccination; outcome: MIS-C, as per the CDC/WHO case definition or ICD-10 diagnosis codes for MIS-C (DB972B and DB972B1). Search terms were combined and are shown in Supplementary Data S1.

LvdG and MHS were in charge of the systemic literature review, removal of duplicates, independently reviewing abstracts of each reference to identify articles for a full review, reviewing the full articles of eligible abstracts and determined each article’s eligibility for inclusion independently. Discrepancies were resolved by consensus, with additional input from MJ as required. The search was performed in June and July 2022. Searched databases included Medline (via PubMed) and Embase. The list was further extended by reviewing the reference lists of identified papers to check for studies that might have been missed in the search strategy, i.e. the “snowballing” method. All original studies, including cohort studies, surveys and surveillance studies published up to June, 2022 were eligible for inclusion. Exclusion criteria were case-report and case series studies, literature or systematic reviews, non-English papers, papers focusing on adults or patients with comorbidities, articles focusing on MIS-C without reference to vaccination, and articles with other primary endpoints. Papers focusing on the effect of vaccination on SARS-CoV-2 infection rather than MIS-C were excluded as well. LvdG and MJ assessed the quality of the included studies.

### Meta-analysis

A meta-analysis was performed in order to quantify the effect of COVID-19 vaccination on the occurrence of MIS-C. For this analysis, only nationwide observational studies without overlapping cases of MIS-C were eligible. If missing from the included studies, frequency of age-matched vaccinated and unvaccinated children without MIS-C were obtained from national COVID-19 dashboards and censuses for similar time periods [21–26]. Pooled odds ratios (OR) with corresponding 95% confidence intervals (CI) were calculated using a common (i.e. fixed) and random effects model with Mantel-Haenszel weighting. Heterogeneity of included studies was quantified using the I^2^ and τ^2^ measures and tested using the Cochran’s Q test. Publication bias was visually assessed using a funnel plot.

## Results

### Included studies

The systemic literature review yielded 13 studies that met the criteria and these were included for data extraction. The studies included two case-control studies, two randomized controlled trials (RCTs), two surveillance studies, two cohort studies, one project report, one survey, one risk-benefit analysis (outline of a CDC presentation), one brief report and one editorial letter. The brief report and editorial letter were used for background information since no new results were presented in these articles. Most studies researched the original strain vaccine during the COVID-19 Delta variant dominant era, but some studies compared results from this period time with cases during a period dominated by the Omicron variant.

**Fig. 1:**
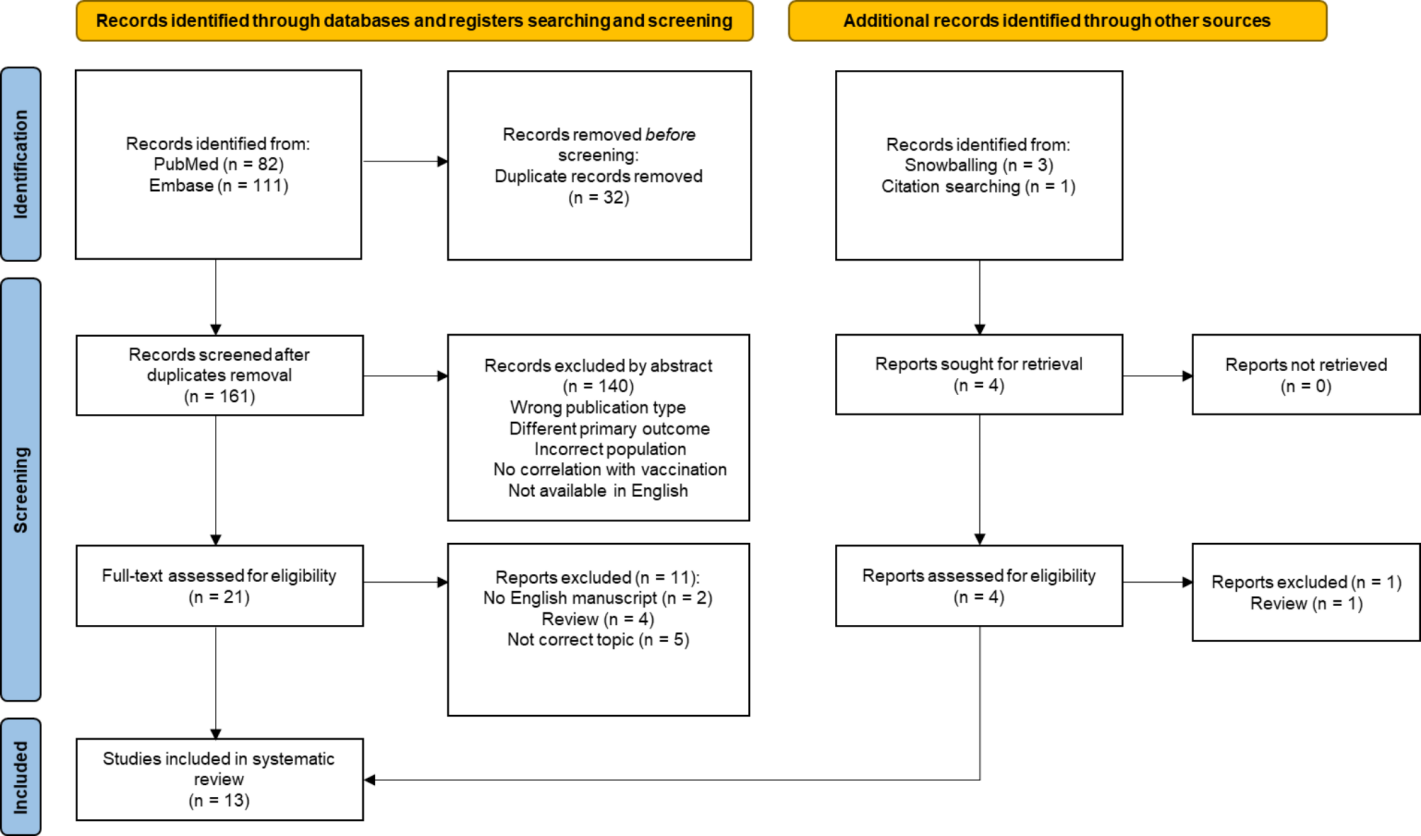
PRISMA flow diagram for the search of databases

The PRISMA flow chart for this study is shown in Fig. 1. A summary of all the included studies including the quality of the studies, using standardized critical appraisal criteria and levels of evidence of the Oxford Centre for Evidence-Based Medicine [27], is shown in Table 1.

**Table 1:** Characteristics of the studies selected in the systematic review and meta-analysis

	Authors	Date	Study Design	Vaccine	Viral variant	Study population	Patients (age)	Adverse events	Level of evidence: preventing MIS-C	Level of evidence: safety
Studies focusing on effectiveness of COVID-19 vaccination against MIS-C.										
**1**	Zambrano et al.(29)	2022, January	Case-control study	BNT162b2 (Pfizer-BioNTech); 2 doses	Delta	102 MIS-C cases, 5 fully vaccinated, 97 unvaccinated. United States.	12–18 years	-	2B	NA
**2**	Levy et al.(28)	2021, December	Cohort study	BNT162b2 (Pfizer-BioNTech; > 95%), mRNA-1273 (Moderna; < 5%), and other COVID-19 vaccines (< 1%)	Delta	33 MIS-C cases, none fully vaccinated, 7 partially vaccinated, 26 unvaccinated. France.	12–18 years	-	2B	NA
**3**	Nygaard et al.(30)	2022, May	Cohort study	BNT162b2 (Pfizer-BioNTech)	Delta	52 MIS-C cases, 1 fully vaccinated, 52 unvaccinated. Denmark.	Unvaccinated: < 18 years Vaccinated: 12–18 years	-	2 C	NA
**4**	Miller et al.(32)	2022, June	Case-control study	mRNA vaccine	Delta; Omicron	763 MIS-C cases, 23 fully vaccinated, 40 partially vaccinated, 697 unvaccinated. United States.	5–20 years	-	2B	NA
**5**	Oliver et al. (39,48)	2021, December	Risk-benefit analysis	BNT162b2 (Pfizer-BioNTech)	-	130 MIS-C cases prevented. United States.	5–11 years	-	2 C	NA
**6**	Stein et al.(34)	2022, January	Project report	BNT162b2 (Pfizer-BioNTech)	-	220 MIS-C cases, 1 fully vaccinated. Israel.	Unvaccinated: < 18 years Vaccinated: 12–18 years	-	2 C	NA
Studies focusing on MIS-C as a possible side effect of COVID-19 vaccination.										
**7**	Ouldali et al. (36)	2022, April	Population-based surveillance	BNT162b2 (Pfizer-BioNTech); mRNA-1273 (Moderna)	-	12 cases of hyperinflammation syndrome.	12–17 years	-	2 C	NA
**8**	Yousaf et al. (35)	2022, February	Surveillance study	BNT162b2 (Pfizer-BioNTech); mRNA-1273, Ad26.COV2.S	Delta	1 MIS-C case per million individuals receiving one or more doses.	12–20 years	-	2 C	NA
**9**	Hoste et al. (37)	2022, May	Cross-sectional survey	mRNA vaccine	-	273 patients with a MIS-C history, no relapse.	-	No relapses in individuals with history of MIS-C.	2 C	NA
Studies about COVID-19 vaccine safety, immunogenicity and efficacy.										
**10**	Creech et al.(3)	2022, May	RCT	mRNA-1273 (Moderna)	Delta	-	6–11 years	No cases of MIS-C were reported.	NA	1B
**11**	Walter et al.(2)	2022, April	RCT	BNT162b2 (Pfizer-BioNTech)	Delta	-	5–11 years	No cases of MIS-C were reported.	NA	1B
Other.										
**12**	Curatola et al. (45,49)	2022, February	Brief report	-	Omicron	-	-	-	-	-
**13**	Mangat et al. (38)	2022, March	Editorial letter	-	Omicron	-	-	-	-	-

### Efficacy of current vaccines in preventing MIS-C development

Recent research by Levy et al. in France and Zambrano et al.in the United States showed, in a cohort study and case-control study respectively, that COVID-19 mRNA vaccination is associated with a lower incidence of MIS-C in children aged 12–18 years compared to unvaccinated and incompletely vaccinated children [28, 29].

Levy et al. reported a hazard ratio for the risk of MIS-C of 0.09 (95% CI: 0.04–0.21) for adolescents after the first vaccine dose compared with unvaccinated adolescents. Pediatric patients diagnosed with MIS-C were included during the Delta wave, between September 1 and October 31, 2021. The vaccine status of patients was known; >95% were vaccinated with BNT162b2 (Pfizer-BioNTech), < 5% with mRNA-1273 (Moderna) and < 1% with other COVID-19 vaccines. During the observational period, 107 MIS-C cases were reported. Among them were 33 (31%) adolescents eligible for vaccination with median age of 13.7 years. None of these children were fully vaccinated (i.e. received two doses), seven children received one dose and 26 children with MIS-C were unvaccinated [28]. Similar results were observed in the study by Zambrano et al. They conducted their research in the era that the Delta variant of SARS-CoV-2 was most prevalent as well, from July 1 to December 9, 2021. The adolescents median age was 14.5 years and 102 MIS-C cases were reported; 95% of the cases had not been vaccinated and 5% were fully vaccinated children. Importantly, 37% of the MIS-C patients, all of them unvaccinated, needed life support including invasive mechanical ventilation, vasoactive infusions and extracorporeal membrane oxygenation (ECMO). Authors concluded that two doses of the Pfizer-BioNTech vaccine lead to a protective effect against MIS-C with an effectiveness of 91% [29].

Nygaard et al. aimed to determine the incidence of MIS-C in the Danish population after infection during the Delta era in children aged 0–17 years old between August 1, 2021 and February 1, 2022 [30]. During this period, 52 MIS-C cases were reported. One MIS-C patient was fully vaccinated among 9,855 vaccinated individuals and 51 patients were unvaccinated among 175,458 unvaccinated individuals infected with SARS-CoV-2. Of these patients, 48 had PCR-confirmed SARS-CoV-2 infection with the Delta variant. Based on these observations, the incidence of MIS-C in unvaccinated individuals was estimated to be 1 in 3,400 unvaccinated children (183 per 1,000,000 unvaccinated person-years) compared to an incidence of MIS-C in fully vaccinated children of 1 in 9,900 individuals (11 per 1,000,000 vaccinated person- years). The rate of vaccination in October 2021 was 72% for children aged 12–15 years and 88% for children aged 16–17 years in Denmark [31]. Nygaard et al. concluded that the vaccine efficacy against MIS-C is 94% [30], comparable with the findings of Zambrano et al.

Miller et al. aimed to compare the incidence of MIS-C cases during Delta and Omicron circulation in the United States in vaccinated and unvaccinated children [32]. A total of 3,554 patients were reported between October 18, 2020 and July 8, 2021 during the third, Delta predominant, wave. However, during the fourth wave, when Omicron was in circulation between July 9, 2021 and January 31, 2022, a total of 2,116 MIS-C cases were reported. This was a clear reduction in incidence, and during this wave, the proportion of patients aged 5–11 years was significantly higher compared with the third wave (p < 0.001). This could be explained by a protective effect of the vaccine, which was given to children aged 12–18 years during the Delta wave, or merely the new Omicron variant. Overall, 763 MIS-C patients aged 5–20 years were eligible to be assessed for full vaccination status before MIS-C onset; 697 (91%) of these patients had no vaccine doses reported and 23 (3.0%) were fully vaccinated. ICU-level care was reported in a higher proportion of patients with no vaccination compared to fully vaccinated patients (61% vs. 48%). All 15 patients who received ECMO and 17 who died were reported to be non-vaccinated [32].

Additionally, a recent study of MIS-C in Israel in which 6% and 15% of patients in the Delta and Omicron waves, respectively, had received two doses of COVID-19 vaccine at least two weeks before admission, reported that MIS-C incidence was lower during the Omicron wave compared with the Alpha and Delta variant waves, and the proportion of cases with ICU admission decreased during successive waves [33].

### Meta-analysis of protective effect

**Fig. 2:**
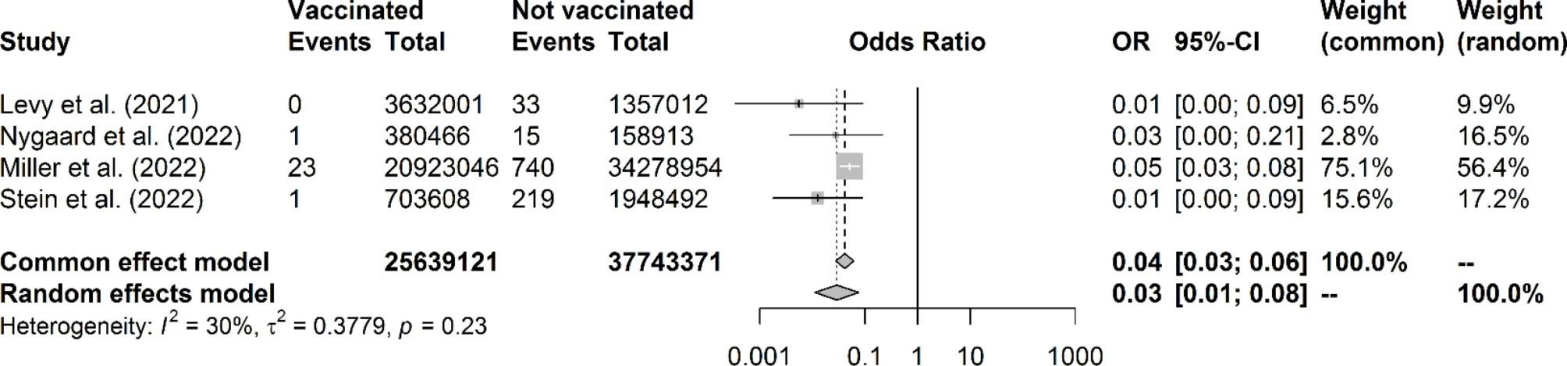
Forest plot of meta-analysis for the effect of COVID-19 vaccination on the number of MIS-C events. Boxes around study effect estimates reflect relative weight in the analysis. P-value reflects Cochran’s Q test of heterogeneity. CI: confidence interval, OR: odds ratio

**Fig. 3:**
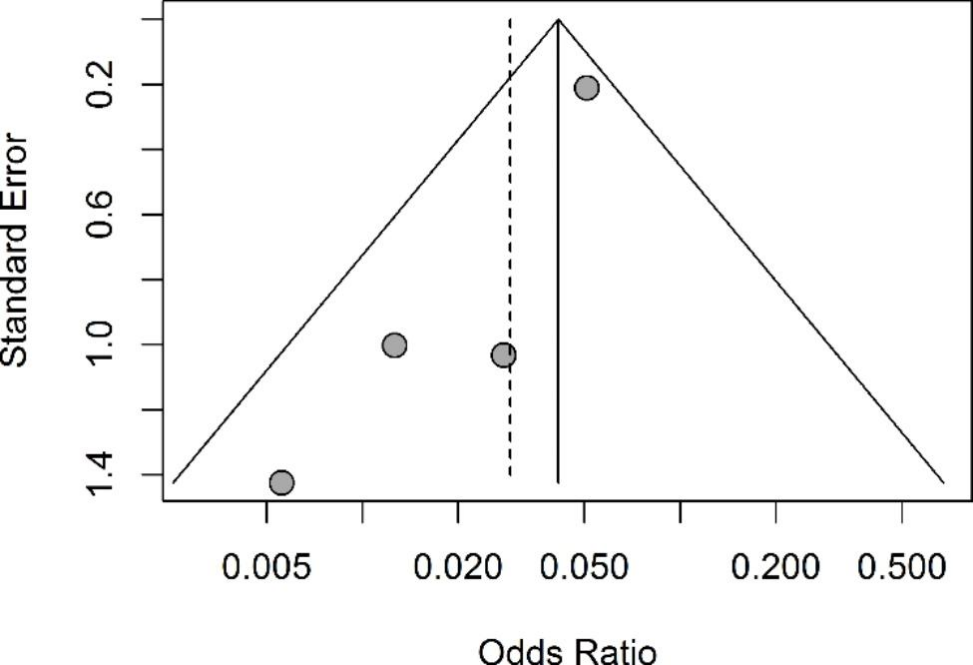
Funnel plot of studies included in meta-analysis. Vertical line indicates pooled common effects estimate. Vertical dashed line indicates pooled random effects estimate

Four studies were eligible for a meta-analysis on the effect of COVID-19 vaccination on MIS-C development (Fig. 2). Frequency of vaccinated and unvaccinated children without MIS-C were obtained from national COVID-19 dashboards and censuses for the studies of Nygaard et al. (age group: 10–17 years old), Miller et al. (age group: 5–17 years old) and Stein et al.[34] (age group: 0–19 years old) [30, 32, 34]. These data are provided in Supplementary Table S1. The pooled OR for MIS-C in vaccinated children compared to unvaccinated children was 0.04 (95% confidence interval: 0.03–0.06). Pooled ORs were significantly protective in both the common and random effects model. There was no indication for heterogeneity of included studies (p = 0.23). The funnel plot was asymmetric indicating a possibility of publication bias (Fig. 3).

### Safety data on COVID-19 vaccination as a trigger for MIS-C development

The previously discussed studies have concluded that COVID-19 mRNA vaccines are safe and effective in preventing MIS-C in children aged 12–18 years. Because MIS-C is considered an auto-immune or abnormal immune reaction after a SARS-CoV-2 infection, it is of great importance to monitor whether MIS-C can occur after COVID-19 vaccination as well.

A recent study by Yousaf et al. stated, following an integrated surveillance study in the United States with data from December, 2020 - August, 2021, that MIS-C after a COVID-19 vaccination is very rare [35]. Using two surveillance systems, they identified 21 MIS-C cases. The mean age of the patients was 16 years and 62% were male. Fifteen MIS-C patients received BNT162b2 (Pfizer-BioNTech) and ten of them had only received one dose before disease onset. The median time between the last injection and disease onset was eight days when children had received one dose (11 MIS-C cases), and five days when they were fully vaccinated (10 MIS-C cases). Importantly, 15 out of these 21 patients had evidence of a past or recent SARS-CoV-2 infection [35].

Similar observations were seen in France [36]. A post-authorization national population-based surveillance study was performed in children aged 12–17 years with data from June, 2021 till January, 2022. During this time, 102 cases of myocarditis or pericarditis and 12 cases of MIS-C were reported as the authors agree that those are likely different entities. The median age of the patients was 12 years; 83% were male. Ten patients had received BNT162b2 (Pfizer-BioNTech) and two patients had received mRNA-1273 (Moderna). The median time from vaccination to the onset of illness ranged from 1 to 42 days. Six cases occurred following the first dose and six following the second dose. In the French study, 4 out of 12 patients had evidence of a recent SARS-CoV-2 infection. The reporting rate of MIS-C was 2.9 per 1,000,000 vaccinated children aged 12–17 years. To compare, a MIS-C rate of 113.3 per 1,000,000 matched age infected children was determined. Furthermore, a lower rate of PICU transfer was seen for post-vaccine MIS-C (4/12, 33%) compared to post-SARS-CoV-2 MIS-C (143/199; 72%). It was concluded that the risk of MIS-C as a side effect of the mRNA vaccine is much lower than the risk of MIS-C post-infection, and very few vaccine-related adverse events were reported [36].

In addition, data from Hoste et al. from an international cross-sectional survey showed that children who had a history of MIS-C could be vaccinated safely and effectively. Two hundred seventy-three patients with a history of MIS-C were vaccinated with mRNA vaccine and all reported cases did not report relapses of MIS-C or other severe inflammatory side effects after SARS-CoV-2 [37].

## Discussion

In this systemic literature review and meta-analysis, the effect of the mRNA COVID-19 vaccine on the development of MIS-C has been addressed. This systematic review lends supportive evidence that vaccination of children and adolescents is highly associated with reduced MIS-C development and underscores the importance of vaccination of all eligible children. In our meta-analysis, we found that the pooled OR for MIS-C in vaccinated children compared to unvaccinated children was 0.04 (95% confidence interval: 0.03–0.06).

Studies conducted in France and the United States during the Delta wave have shown a reduced incidence of MIS-C after mRNA COVID-19 vaccination in children aged 12–18 years, with a hazard ratio for the risk of MIS-C of 0.09 after one vaccine dose compared with unvaccinated adolescents in France [28]. Two doses of the Pfizer-BioNTech vaccine led to a protective effect against MIS-C with an effectiveness of 91% in the US cohort [29]. In addition, in Denmark, the Pfizer–BioNTech vaccine was protective against MIS-C in 94% during the Delta wave [30].

Altogether, it can be concluded that the protective effect of vaccination outweighs the triggering effect of vaccination on MIS-C, as the risk of developing MIS-C as an adverse effect of vaccination is much lower (2,9 per 1,000,000 children), compared to the risk of MIS-C post-infection (113 per 1,000,000 children) [36]. Furthermore, based on the literature so far, it remains unknown if COVID-19 vaccination really can cause MIS-C or whether vaccinated children who developed MIS-C had a recent (sometimes unnoticed) SARS-CoV-2 infection.

It is noteworthy that the conclusions from our systemic review are based mostly on children in the age category of 12–18 years old. On October, 2021 the Pfizer-BioNTech vaccine was approved by the FDA for children aged 5–11 years [2, 3]. Therefore, the preventive effect seen in adolescents has not yet been evaluated in these younger children [38]. In 2021, the CDC mentioned that MIS-C was most frequent among children 5–11 years, with 2,316 cases of MIS-C reported in the United States at that time [39]. However, a plausible explanation for this would be that adolescents were already vaccinated during this period and since this has a protective effect, the number of MIS-C patients among the younger children increased. Based on a benefit-risk analysis from the CDC using recent epidemiological data for both 5–11 years old males and females, 130 MIS-C cases could be prevented for every million Pfizer-BioNTech vaccinations. Thus, the number needed to vaccinate (NNV) was 1,1226, indicating that in order to prevent one MIS-C case in children aged 5–11 years, 1,1226 children should be vaccinated [39]. In addition to avoiding the development of MIS-C, COVID-19 vaccination also decreases the morbidity and mortality associated with the disease with low numbers of adverse events associated with the vaccination [40, 41]. In the upcoming months, data should be gathered to make a similar evidence-based conclusion about vaccination and MIS-C in children younger than 12 years.

The exact pathophysiology of MIS-C remains unknown, but recent research showed that patients with MIS-C have a different type of host immune response compared to patients with acute COVID-19. This immune response includes superantigen-like activation of T-cells with expansion of polyclonal expansion of TCR Vbeta 21.3^+^ CD4^+^ and CD8^+^ T-cells, something which is not seen in toxic shock syndrome, Kawasaki disease or COVID-19 in general. In addition, host genetics might alter the susceptibility to develop MIS-C [16–19, 42–44].

Possible explanations for a protective mechanism of the vaccine on MIS-C include the prevention of COVID-19, since MIS-C is a complication of this disease and the vaccine does not seem to induce the super-antigen driven T-cell clone, whereas the wildtype infection does. A second hypothesis is that the vaccine could have a direct immune protective effect, preventing a hyper inflammatory condition [30, 44].

The epidemiology of the COVID-19 virus is changing constantly, and the Omicron variant may lead to a milder disease course but might be also more contagious [38]. It is not known yet if the Omicron variant is more or less associated with MIS-C compared to other variants [45]. Future COVID-19 variants may affect the clinical course and may influence the incidence of MIS-C cases.

The studies included in this systematic review were conducted during a period when the Delta variant was dominant and Omicron was looming [38]. The question arises, whether the conclusions that were made about the Delta era can be translated to the Omicron era. The CDC reported that Omicron has a higher transmission rate [38]. Since Omicron has become dominant, researchers have focused on comparing COVID-19 and MIS-C cases between the Delta and Omicron era. Following an observational retrospective study that was performed in Rio de Janeiro with children aged 0–18 years, it was concluded that fewer hospital admissions occurred during the Omicron wave [46]. Besides an increased number of children with acquired immunity due to prior (asymptomatic) SARS-CoV-2 infection and the fact that the Omicron variant is less pathogenic, another explanation for this reduction in hospital admissions could be the increased number of vaccinated children. Miller et al. aimed to compare the incidence of MIS-C cases during the Delta and Omicron waves in the United States and found a clear reduction in incidence and less often severe organ damage during the latter wave [32]. However, they observed an increase in children aged 5–11 years with MIS-C. Similar observations were observed in Israel [33]. This could again indicate that the COVID-19 vaccine, which was given to children aged 12–18 years during the Delta wave, has a protective effect on developing MIS-C. These findings might also indicate the importance of COVID-19 vaccination in children aged 5–11 years, although the number of studies in the current systematic review that included this age group was limited. Other possible explanations for the reduced number of (severe) MIS-C cases during the Omicron wave include lower pathogenicity of the Omicron variant, prior SARS-CoV-2 infection and improvement in the treatment of MIS-C.

This systematic review has some limitations. These findings reflect the current reality in the complicated situation of a global pandemic, multiple variant outbreaks (Omicron variant had not been studied in most of the articles) and continuing vaccination processes all over the world with gradual addition of further age groups. Furthermore, the main limitation of the included studies is their small group size and heterogeneous study designs. However, MIS-C is a rare complication and the small group size can therefore still be seen as realistic, especially because the studies are based on national data which enhances statistical power. Another limitation concerns the virus variant, as the studies were conducted during the SARS-CoV-2 Delta wave. It has not yet been investigated whether the mRNA vaccine will also show a high vaccine efficacy against MIS-C during the SARS-CoV-2 Omicron wave or other future variants, and this also cannot be concluded with certainty from this systematic review. This generalization might be complicated even further since an increased number of children around the world will have currently already experienced a SARS-CoV-2 infection. Besides the low number of patients in some studies, there were few reports about regional variation and individual risks factors for MIS-C, such as sex, race/ethnicity, and comorbidities. Miller et al. mention a predominance of males in MIS-C and an increasing proportion of non-Hispanic White and non-Hispanic Black patients during successive COVID-19 waves [32]. The studies by Levy et al., Zambrano et al., and Nygaard et al. also report a male to female predominance in MIS-C (of 81%, 71% and 73%, respectively) [28–30].This analysis was not designed to evaluate waning immunity or duration of protection against MIS-C. Regarding the safety profile studies, there could be some bias related to recall or notification of adverse events. Nevertheless, it seems unlikely that healthcare providers would not have been notified of important inflammatory complications or MIS-C relapses. Regarding the meta-analysis, not all studies reported information about the number of vaccinated and unvaccinated children without MIS-C and these data had to be collected from national dashboards and censuses. This resulted in some slight discrepancies between age groups of children with and without MIS-C and hence we could not present risk ratios and a NNV, but merely ORs. Also, the funnel plot indicated a possibility of publication bias, but because of the low number of included studies this is uncertain. Nevertheless, the forest plot, I^2^ statistic and Cochran’s Q test indicated no heterogeneity of study results and we performed both fixed and random effects meta-analyses with similar results. Finally, it is well possible that some reported cases of MIS-C after a COVID-19 vaccination were related to unnoticed or undiagnosed SARS-CoV-2 infections with false negative infectious investigations.

This review could become an important contribution for discussion whether or not to vaccinate children against COVID-19. Eventually, the review can help to increase international COVID-19 vaccination rates in children from the age of 5 years. Currently, in the United States, only 29% of eligible children have been fully vaccinated in the age group of 5–11 years, and 59% in the age group of 12–17 years [47].

In the coming period, data collection will have to be continued in order to monitor the incidence of MIS-C, the vaccine efficacy in younger children (< 12 years old) and possible side effects. The epidemiology of SARS CoV-2 infection is changing rapidly and a possible new wave may present. With the small number of cases of MIS-C globally, establishing an international research collaboration would be of great value to combine studies and data effectively [5]. Finally, more research will be needed to compare MIS-C cases triggered by the Delta, Omicron, and possible future unknown variants [45].

## Conclusions

Our systematic literature review and meta-analysis indicate that COVID-19 mRNA vaccines in children are safe and associated with significantly less development of MIS-C during the Delta wave. MIS-C after COVID-19 vaccination in children is rare and significantly less common than after SARS-CoV-2 infection in the same age-group. These findings form the most comprehensive review and analysis of the available evidence, further reinforcing the recommendation for COVID-19 vaccination children, and could greatly contribute to evidence-based guidelines. Still, further larger studies are needed to make an evidence-based statement on the protective effect of COVID-19 vaccination on developing MIS-C, addressing children < 12 years of age and comparing MIS-C cases triggered by the Delta, Omicron and other SARS-CoV-2 variants.

### Electronic supplementary material

Below is the link to the electronic supplementary material.

Supplementary Material 1

## Data Availability

All relevant data are reported in the article. Additional details can be provided by the corresponding author upon reasonable request.

## Abbreviations

CDC: Centers for Disease Control and Prevention
COVID-19: coronavirus disease 2019
ECMO: extracorporeal membrane oxygenation
EMA: European Medicine Agency
FDA: Food and Drug Administration
MIS-C: multisystem inflammatory syndrome in children
NNV: number needed to vaccinate
PRISMA: Preferred Reporting Items for Systematic reviews and Meta-Analysis
SARS-CoV-2: severe acute respiratory syndrome coronavirus 2
